# Activation of transient receptor potential channel Sm.(*Schistosoma mansoni*)TRPM_PZQ_ by PZQ, enhanced Ca^++^ influx, spastic paralysis, and tegumental disrupture—the deadly cascade in parasitic schistosomes, other trematodes, and cestodes

**DOI:** 10.1007/s00436-020-06763-8

**Published:** 2020-06-30

**Authors:** Achim Harder

**Affiliations:** WE Biology, Heinrich-Heine-University, Düsseldorf, Germany

**Keywords:** Praziquantel, Sm.TRPM_PZQ_, Calcium homeostasis, Contraction, Tegument disruption, HII structures, Worm death

## Abstract

After almost 50 years of praziquantel (PZQ) research, Park and Marchant (Trends Parasitol 36:182–194, 2020) described the Ca^++^-permeable transient receptor potential (TRP) channel Sm.TRPM_PZQ_ in *Schistosoma mansoni* as target of PZQ. Here we describe the deadly cascade in schistosomes which is induced by the (R)-PZQ enantiomer that includes contemporaneous stereoselective activation of Sm.TRPM_PZQ_-mediated Ca^++^ influx, disturbed Ca^++^ homeostasis, Ca^++^-dependent spastic paralysis, and Ca^++^- and PZQ-dependent disruption of parasitic teguments. Under normal conditions, there is a reversible balance between bilayer, isotropic, and HII phases in biological membranes (Jouhet 2013). In vitro, we could observe an irreversible but not stereoselective transition to the HII phase in liposomes consisting of phosphatidylethanolamine (PE) and phosphatidylserine (PS), two naturally occurring phospholipids in schistosomes, by the concerted action of Ca^++^ and PZQ (Harder 2013). HII structures are a prerequisite for induction of fusion processes (Jouhet 2013), which, indeed, become visible as blebs, vacuolation processes, and large balloon-like surface exudates in a large variety of PZQ-sensitive parasitic flukes and cestodes after PZQ treatment. These tegument damages are irreversible. As homologs of Sm.TRPM_PZQ_ are also present in the other trematodes *S. japonicum*, *S. haematobium*, or *Clonorchis sinensis* and cestodes *Taenia solium*, *Echinococcus multilocularis*, or *Hymenolepis microstoma* (Park and Marchant, Trends Parasitol 36:182–194, 2020), it is suggested that a similar deadly cascade will be operating generally in PZQ-sensitive parasites.

## Introduction

Praziquantel (PZQ), the drug of choice against a variety of blood, liver, lung, intestinal flukes, and cestodes in human and veterinary medicine, had been discovered by a joint cooperation between Bayer AG and E. Merck (Darmstadt) in 1972 (Andrews et al. 1983; Harder 2002). E. Merck worked on vasoactive compounds for blood pressure regulation in humans (Seubert 1976). In a patent application, the Merck inventors claimed the synthesis of 3-Acyl-4-oxo-pyrazino-isochinolin-derivatives as anthelmintics against trematodes and cestodes. However, the class of pyrazinoquinolines was initially to be used in a completely different medicinal field as also as psychotropic and blood pressure-influencing compounds. At the beginning of the 1970s, the search for effective tranquilizers with few side effects was still underway in the pharmaceutical industry (Groll 1984). Indeed, Gunaratne et al. (2018) describe PZQ as a drug with polypharmacologal properties and emphazise its host vascular effectivity.

In the field of chemotherapy against human schistosomiasis, PZQ is useful for individual and mass treatment because of its high efficacy, excellent tolerability, and simple way of application (Cioli et al. 2012). The anthelmintic activities against trematodes and cestodes of PZQ have been reviewed in a large variety of publications over a long period of time. But here we rely on only some basic references (Andrews et al. 1983; Andrews 1985; Cioli et al. 2012; Cupit and Cunningham 2015; Harder 2013).

In veterinary medicine, PZQ is constituent in a large variety of useful so-called allwormer combinations with different antinematodal drugs, e.g., benzimidazoles, imidazothiazoles, tetrahydropyrimidines, macrocyclic lactones, or cyclic octadepsipeptides, thereby extending the anthelmintic sprectrum by including cestodes (Deplazes et al. 2013a, 2013b).

Thus, PZQ is one of the two constituents of the dewormer Profender® besides emodepside. While emodepside exerts its anthelmintic activities against nematodes by opening the SLO-1 channel and/or modifies its normal voltage current or Ca^++^ dependence, PZQ is responsible for the activities against cestodes (Krücken et al. 2012). However, the mode of action of PZQ is still enigmatic despite many studies (Cupit and Cunningham 2015).

The recently described Sm.TRPM_PZQ_, the transient receptor potential (TRP) channel of *S. mansoni*, belongs to a member of the transient receptor potential melastatin (TRPM) channel subfamily and is broadly expressed in PZQ-sensitive flatworms (Park and Marchant 2020). With a log *P* value of 2.3–2.5, this hydrophobic drug will preferably interact with hydrophobic regions of receptors/channels and membranes of worms. Therefore, Sm.TRPM_PZQ_ is an excellent candidate for being the PZQ receptor, because it becomes activated by PZQ. Beside this receptor, the focus of this review is also to summarize PZQ-phospholipid-interactions, which are suggested to play an important role in the PZQ-evoked disruption of parasitic tegument membranes in connection with PZQ-evoked Ca^++^ influx through the pore of Sm.TRPM_PZQ_.

## PZQ resistance

Despite long wide usage of PZQ in human and in veterinary medicine, there has not been evidence for resistance against this drug in schistosomiasis up to now (Doenhoff et al. 2008; Wang et al. 2012; Cupit and Cunningham 2015). In cases of Senegalese population heavily infected with *S. mansoni*, the view is now that the intensity of infection and timing of follow-up period, which left enough time for the development of PZQ-insensitive, prepatent infections, were more significant factors (Cupit and Cunningham 2015). There are also studies from Egyptian and Kenyan patients harboring *S. mansoni* with significantly less sensitivity to PZQ. However, the worms showed either diminished reproductive fitness or proved difficult to maintain in the laboratory beyond one or two generations (Cupit and Cunningham 2015). Schistosomes with a decreased sensitivity to PZQ have shown “resistance” at an always modest level and moreover this was often unstable and could not be increased by repeated drug pressure (Doenhoff et al. 2008).

In summary, although minor variations of PZQ sensitivity occur, they are at present not at a level that may threaten clinical efficacy, and there is no evidence that any “PZQ resistance” is spreading in the field (Doenhoff et al. 2008; Cupit and Cunningham 2015).

In the laboratory, PZQ-resistant schistosome strains have been generated by using increasing sublethal and then previously lethal doses of PZQ over several generations in mice. Even these strains with reduced sensitivity lack long-term stability (reviewed by Cupit and Cunningham 2015). These authors suggest ATP-binding cassette (ABC) proteins may be involved in the molecular mechanism of resistance in field/laboratory isolates.

The urgency for new antischistosomal and anticestodal drugs is seen by the recent alarming appearance of PZQ-resistant zoonotic *Dipylidium caninum*, infecting dogs, cats, and humans (Chelladurai et al. 2018. During the time course from 2016 to 2018, cases of clinical resistance in dogs with this cestode against PZQ and the structurally related epsiprantel have been reported.

## Pharmacokinetics of PZQ

Only those pharmacokinetic properties which seem relevant for the mechanism of action of PZQ are mentioned here. Commercially available PZQ is a racemic mixture of two enantiomers (Andrews et al. 1983; Andrews 1985; Park and Marchant 2020). (R)-PZQ, the laevo-enantiomer, exerts high anthelmintic activities against *S. mansoni* in vivo or in vitro, *S. haematobium* in vitro or *Hymenolepis nana*, while (S)-PZQ, the dextro-enantiomer, is far less anthelmintically active (Andrews et al. 1983; Andrews 1985; Kovac et al. 2017; Park and Marchant 2020). Interestingly, (S)-PZQ harbored high anthelmintic activity against *S. haematobium* in Syrian hamsters (Kovac et al. 2017).

Between 80 and 100% of PZQ are absorbed from the intestine after oral application very rapidly (Andrews 1985). Maximal serum concentrations are reached within 1 h in all animal species investigated (Andrews 1985). PZQ, reaching the liver via the portal vein, is metabolized extensively at a high rate (Andrews 1985). The major metabolite in serum of man and animals is the 4-hydroxycyclohexylcarbonyl analogue of PZQ (Andrews 1985; Kovac et al. 2017). PZQ is distributed rapidly throughout the body.

In mice the drug is very rapidly distributed throughout all organs. The PZQ concentrations in lungs, pancreas, adrenals, pituitary, and salivary glands are higher than in the plasma. Only low concentrations of PZQ are found in the brain (Andrews et al. 1983; Andrews 1985).

Moreover, PZQ is reversibly bound to plasma proteins, the portion of protein-bound drug is 76% (Andrews et al. 1983).

PZQ modulates host vascular tone in blood vessels where adult schistosomes reside (Gunaratne et al. 2018). In resting mesenteric arteries, PZQ causes a constriction of basal tone. This effect is mediated by (R)-PZQ activation of the endogenous serotoninergic G protein-coupled receptor (GPCR) 5HT_2B_R. However, when mesenteric vessels are precontracted by high K^+^-evoked depolarization, a novel vasodilatory action of PZQ is observed. Interestingly, this relaxant effect of PZQ in those mesenteric vessels was caused by (S)-PZQ and not by (R)-PZQ and mimicked by TRPM8 agonists. Therefore, it was concluded that (S)-PZQ is vasoactive over a micromolar range in mesenteric arteries. The molecular mediators of this effect remain to be identified (Gunaratne et al. 2018).

## Pharmacodynamics of PZQ

In vitro studies using ^14^C-PZQ have demonstrated that *S. mansoni* worms take up PZQ rapidly from protein-free media (Andrews 1985). The concentration of the drug inside the parasite is equal to that in the medium within 2 min and increased further with time. Drug uptake was found to be reversible: 93% of the ^14^C-radioactivity was lost from schistosomes after transfer to drug-free medium within 30 min. ^14^C-radioactivity was equally distributed throughout the parasite tissues. Furthermore, parasites are unable to metabolize PZQ. The uptake of PZQ was not affected by the removal of Ca^++^ from external medium (Xiao et al. 1984). Studies with *S. japonicum* revealed similar results for incubation periods up to 8 h (Xiao et al. 1981). However, drug distribution in the parasite tissues was not uniform. Minimal and maximal amounts of ^3^H-radioactivity, which allows a better resolution than the ^14^C-label, were detected by autoradiography in the testes of the males and the vitelline gland of the females, respectively.

In rats treated with a dose of 300 mg/kg PZQ, drug concentrations of 2 μM were found in the peripheral venous blood, while 7 μM were measured in the portal blood (Andrews 1985; Chan et al. 2017). This means 3–5 times higher PZQ levels are present in the portal veins compared to that in peripheric blood vessels for *S. mansoni* and *S. japonicum* in vivo.

To get insight into the mode of PZQ action the knowledge of threshold concentrations of the drug is necessary, at which biochemical PZQ effects occur (Andrews 1985; Harder 2013). As PZQ metabolites formed by the mammalian host exert only minor antischistosomal activities, only unchanged PZQ is responsible for the mechanism of action. From simultaneous studies of pharmacokinetics and therapeutic efficacies of PZQ in animal models, the threshold concentration is estimated to be in the range of 0.6 up to 1 μM, and blood levels of about 0.6 μM PZQ prevailed for about 4 h (Andrews 1985). These conditions are met in host animals and patients receiving antischistosomal therapy. Therefore, only those effects of PZQ that are induced by concentrations of about 1 μM are likely to be relevant for the understanding of the mechanism of action of PZQ against parasitic trematodes and cestodes (Andrews et al. 1983).

## Properties of schistosome tegument

The tegument of adult schistosomes is a cilialess, cytoplasmic but noncellular, metabolically active layer (Mehlhorn 2008). The functions of the tegument are diverse: protection of the parasite against the host’s immune attack, maintenance of survival, absorption of nutrients, osmoregulation, and excretion. In terms of protection, the parasite incorporates host antigens on its tegument surface and so camouflages itself effectively. The tegument serves as an important site of nutrient uptake and ion and water regulation. It contains various enzymes involved in ATP generation, and it maintains a membrane potential ranging between −35 and −41 mV. The ionic composition of the tegument is different from that of the serum surrounding the parasite. All these phenomena suggest the existence of proton pumps operating in the tegument for maintaining ionic gradients and a pH value of 7.8.

The apical surface of the tegument is a specialized version of the normal phospholipid bilayer membrane, a so-called heptalaminate membrane, which is formed by the stacking of two trilaminate membranes on top of each other. This special adaptation is also known to occur in other blood-dwelling flukes and may be related to the intravascular habitat. The components of tegument underlie rapid rates of turnover. The heptalaminate surface membrane, with a half-life of about 2 to 3 h, appears to be replaced continually by material provided through multilaminate vesicles produced in the cell bodies of the tegument (Mehlhorn 2008).

## Effects of praziquantel on schistosome tegument and muscles

Two striking phenomena are observable in all PZQ-sensitive trematodes and cestodes after exposure to PZQ (Table 1). There is a rapid, surface blebbing (0.32–0.6 μM PZQ), structural damage of the surface (0.5 μM PZQ), and vacuolation as early as 15 min in vivo (Andrews et al. 1983). Moreover, there is an instantaneous tetanic contraction of the musculature. The increase in muscle tension is measurable as half maximal contraction in presence of 1 μM PZQ after 11 s (Table 1; Andrews et al. 1983). In vitro, all these changes occur at concentrations equivalent to therapeutic serum levels at 1 μM within maximal 30 s after contact with the drug (Andrews et al. 1983).

**Table 1:** Cascade of quick PZQ actions including Sm.TRPM_PZQ_ activation, muscle contraction, and tegumental disruption in *Schistosoma mansoni* as a function of time. Data are taken from Andrews et al. (1983) and Park and Marchant (2020)

Organ	Drug concentration	Observed effect	Time after drug exposure of observable effect
First step			
Muscle Sm.TRPM_PZQ_	< 0.6 μM; EC_50_ about 150 nM	Activation of Ca^++^ permeable transient receptor potential channel in *S. mansoni* and disruption of Ca^++^ homeostasis	Direct effect
Instantaneous effects following PZQ binding to Sm.TRP_**PZQ**_			
A. Muscle contraction			
Musculature	0.2 μM	Increase in muscle tension	Seconds
Musculature	1 μM	Half maximal contraction	After 11 s
Musculature	0.32 μM	Tetanic contraction	Seconds
B. Tegumental disruption			
Tegument	0.32–0.6 μM	Surface blebbing	Seconds
Tegument	Around 1 μM	Increased Ca^++^ influx	Seconds
Tegument	0.5 μM	Structural damage of the surface, bleb-like structures	Rapid, instantaneous
Tegument	Around 1 μM	Enhanced uptake of external Ca^++^	Rapid, after 1 min
Tegument	Around 1 μM	Impaired ion fluxes (Na^+^, K^+^), uptake of glucose and adenosine, lactate excretion, glycogen breakdown, impaired activities of membrane associated enzymes: e.g., ATPase	Seconds until minutes
Tegument	0.5 μM	Vacuolation, larger balloon-like exudates	30 s

## Damages of tegumental surface by PZQ

Some experiments, which are performed with intact schistosomes in vitro, revealed that (R)-PZQ was responsible for the PZQ-induced rapid structural damage of the surface observed in vitro (Staudt et al. 1992). However, the intact schistosomes retain intact receptors and tegument membranes. Therefore, the observed stereoselectivity of tegument damage by (R)-PZQ may be due more to the stereoselective binding of drug to Sm.TRPM_PZQ_, present in schistosomes (Park and Marchant 2020). Nevertheless, surface damage is a fundamental action of PZQ visible in vitro and in vivo (Mehlhorn et al. 1981; Bricker et al. 1983; Xiao et al. 1984).

Furthermore, only oral treatment of mice with a curative dose was lethal to the parasites, whereas worms treated with a subcurative or submaximal dose recovered from muscular contraction and the intensive vacuolation (Mehlhorn et al. 1981). The minimum concentration of PZQ required to induce tegument vacuolations is 0.32 μM (Andrews 1985). At PZQ concentrations above 3.2 μM, tegument vacuolations become irreversible and are a function of time of exposure to the drug, rather than of its concentration (Andrews et al. 1983).

Very important, tegument damage by PZQ is only induced in presence of Ca^++^, whereas PZQ-evoked vacuolation does not occur in Ca^++^-free media or in Ca^++^-depleted schistosomes or in the presence of an excess of Mg^++^ (Xiao et al. 1984; Andrews 1985; Park and Marchant 2020).

How tegument damage is brought about cannot be answered by mere morphological and ultrastructural studies. Similar tegument alterations have also been observed in schistosomes exposed to many different conditions such as hypo- or hypertonic media, in vitro culture, hyperimmune serum, complement, major basic protein, lectins, and several antischistosome compounds (Andrews et al. 1983; Andrews 1985). One point, however, is noteworthy. PZQ induces surface blebbing within seconds, while many minutes or hours are required by the other agents.

PZQ causes tegument alterations qualitatively similar to those in schistosomes in a large number of other digenean trematodes living in the lungs, livers, or intestines of their hosts and also in a large variety of cestodes studied (Andrews et al. 1983). The extent of tegument damage may vary between different parasite species. Trematode spp. with an equally thin tegument as in schistosomes, like *Dicrocoelium*, *Opisthorchis*, *Clonorchis*, and *Metagonimus*, react with comparable tegument damage to PZQ (Chai 2013). Only in *Paragonimus westermani* with an exceptionally thick and condensed tegument structure, the PZQ effect is less noticeable, while this effect is absent from the liver fluke *Fasciola hepatica*, which possesses a tegument with an even higher content of fortifying fibrils (Becker et al. 1980; Andrews et al. 1983; Andrews 1985; Chai 2013). Nematodes which possess thick cuticles with a high content of collagen are completely protected against PZQ-induced cuticular damage.

As mentioned above, the surface of the tegument is a complex double bilayer, in which lipids and proteins may be organized into domains characterized by different properties and compositions (Redman et al. 1996). Indeed, there are differences in the distribution and extent of PZQ-induced damage to the surface of different life cycle stages of schistosomes observable. Moreover, domains in the double bilayer surface membranes show different responses to PZQ: in one domain, fluidity was increased, while in the other it was decreased (Lima et al. 1994a, 1994b). These differences in susceptibility to PZQ suggest that membrane composition may be an important factor in the drug’s action (Redman et al. 1996).

## Impaired muscle contraction by PZQ

PZQ-induced effects on schistosome muscles are fast, too: half maximal contraction occurs already 11 s after exposure to 1 μM PZQ (Pax et al. 1978). The PZQ-induced contraction depends on the presence of Ca^++^ ions. Depletion of external Ca^++^ or addition of an excess of Mg^++^ abolishes drug-induced contraction (Redman et al. 1996). Moreover, most inhibitors of known neurotransmitters of *S. mansoni* and other pharmacologically active agents (e.g., carbachol, spiroperidol, bromo-lysergic acid diethylamide, imipramine, dopamine, noradrenaline, 5-hydroxytryptamine, arecoline, metrifonate, pentobarbital, atropine, mecamylamine, or pempidine) do not antagonize this contraction-inducing effect of PZQ. This lack of interaction of PZQ with neuronal sites together with the requirement for Ca^++^ ions has led to the idea that PZQ directly or indirectly affects Ca^++^ flux across biological membranes by interaction with sites not classically associated with the regulation of Ca^++^ transport (Andrews et al. 1983; Andrews 1985).

In addition, it has been shown that 1 μM PZQ induces a rapid increase in the rate of uptake of external Ca^++^ into schistosomes. However, PZQ does not act as an ionophore, although some ionophores (X537 and A23187) were shown to induce contraction in schistosomes (Pax et al. 1978). Also, the Ca^++^ channel blocker D-600 did not prevent PZQ-induced muscular contraction in schistosomes (Fetterer et al. 1980). However, La^3+^, an antagonist of TRP channels, blocks PZQ action on muscular paralysis entirely (Andrews 1985; Park and Marchant 2020). Fluoxetine (FXT), a specific inhibitor of serotonin uptake in mouse brain and in schistosomes, was the only pharmacological agent that interfered with both the influx of Ca^++^ and the induction of musculature contraction (Andrews et al. 1983). Again, all these experiments supported the view that PZQ affects Ca^++^ flux across biological membranes (Andrews 1985).

## Coupling of PZQ uptake, Sm.TRPM_PZQ_-mediated Ca^++^ influx, muscle contraction, and tegument vacuolization

PZQ uptake by *S. mansoni* is the first causative step in a serious of events taking place in the parasites. The drug uptake in protein-free medium is independent on the presence or absence of Ca^++^ (Andrews 1985). The concentration of the drug inside the parasite is equal to that in the medium within 2 min. With a log *P* value of 2.3, PZQ will preferentially bind to hydrophobic sites within all organs of parasites, i.e., it will bind to hydrophobic sites of proteins as well as membranes. Ca^++^ channels in schistosomes were postulated as molecular targets of PZQ resulting in a toxic Ca^++^ influx into the worm (Doenhoff et al. 2008; Cioli et al. 2012). Today it is proposed that the Ca^++^ permeable transient receptor potential (TRP) channel in *Schistosoma mansoni* Sm.TRPM_PZQ_ functions as a link between PZQ and Ca^++^ (Park and Marchant 2020). With an EC_50_ value of about 150 nM, Sm.TRPM_PZQ_ becomes stereoselectively activated by (R)-PRQ accompanied by a sustained cellular Ca^++^ signal in the worms (Park, McCusker, Dosa 2919). The resulting Ca^++^ influx then exerts direct effects on schistosome musclses. Indeed, the time course of responses to PZQ on Sm.TRPM_PZQ_ activation and PZQ-evoked increase of schistosome muscle tension, which occurs within seconds (or less than 1 min) are superimposed. It looks as if both events are coupled directly with each other (Park et al. 2019b; Park and Marchant 2020).

How are responses to PZQ on Sm.TRPM_PZQ_ activation and PZQ-evoked tegument damages coupled to muscular contraction? Concentrations of 3.2 μM PZQ are sufficient to cause tegument vacuolations and surface blebbing in *S. mansoni* within 5 min (Becker et al. 1980). Time course studies revealed that the onset of tegument damages is even more rapid. Surface blebbing is caused within 30 s in presence of 1 μM PZQ (Andrews 1985). Although it is much more difficult to measure these effects exactly, it is surprising how the time course of PZQ-evoked tegument alterations also mirrors the responses of PZQ activation of Sm.TRPM_PZQ_, Ca^++^ influx, and muscle tension.

Redman et al. (1996) supposed that the presence of PZQ-sensitive sites in the surface could explain the susceptibility of the tegument to PZQ-induced damage. Moreover, the tegument is electrically coupled to muscle cells. Therefore, an increase in Ca^++^ levels within the tegument might lead to increased Ca^++^ in sarcoplasmic reticulum which then could induce muscular contraction (Redman et al. 1996). The electrical coupling between tegument and muscle cells would explain why any tegument changes will immediately produce changes in muscular response. This means that PZQ may induce muscle contraction by interacting with tegument rather than affecting muscle cells directly (Redman et al. 1996).

## PZQ is a membrane-active drug

PZQ is known to affect glucose uptake across tegument of *S. mansoni* (Harder et al. 1987a). The same could be observed with FXT and various other amphiphilic cationic drugs, known as “membrane-active” compounds (Seeman 1972). The amphiphilic cationic drugs chlorpromazine, imipramine, amitryptiline, propranolol, and FXT, and the electrically neutral PZQ stimulate glucose uptake and, moreover, lactate excretion in a qualitatively similar way, but quantitatively in a different concentration range. While the five amphiphilic drugs exert a maximal stimulatory effect at concentrations between 10 and 100 μM, PZQ is stimulatory already at 0.1 μM. PZQ is thus much more effective compared to the cationic amphiphilic drugs (Harder et al. 1987b).

Also, the stimulatory effects of serotonin on glucose uptake are completely abolished by PZQ (Harder et al. 1987b). The inhibitory effect of PZQ may be caused by perturbation of the tegument membranes; however, it cannot be excluded that the inhibitory action of PZQ against serotonin-stimulated glucose uptake could rely on a functional antagonistic PZQ action of flatworm 5-HT receptors (Chan et al. 2015). However, PZQ-induced tegument disruption may be responsible for the inhibitory effect of PZQ on two tegument enzyme systems: the ATPase of *S. mansoni* (Nechay et al. 1980) and phosphatases of the cestode *Cotugnia digonophora* (Pampori 1985). This observation cannot be explained by any functional antagonistic PZQ action of flatworm 5-HT receptors.

## Phospholipid composition of schistosome tegument

Unfortunately, tegument phospholipids of *S. mansoni* can only be prepared in very small amounts. Further, the exact composition of these lipids and their distribution across the schistosome tegument are not known. The best approximation is derived from the composition of the multilaminate vesicles, which are contained in the tegument and which are thought to be incorporated into the surface of schistosomes. At least 25% of the total phospholipids of multilamellar bodies of *S. mansoni* are acidic ones: mainly phosphatidylserine (PS) (15%), phosphatidylglycerol (PG) (8%), phosphatidylinositol (5%), and phosphatidic acid. Neutral phospholipids consisting of phosphatidylethanolamine (PE) (25%) and phosphatidylcholine (PC) (28%) are the main fraction (McDiarmid et al. 1982).

## Effects of PZQ on model membranes

Schepers et al. (1988) could show that PZQ is able to destabilize liposomes of synthetic dipalmitoylphosphatidylcholine (DPPC) liposomes by insertion into the bilayer. The PZQ lipid destabilizing capacity is explained by high PZQ lipid interactions and the large area occupied per drug molecule in the lipid layer. It is suggested that PZQ does not modify the lipid structure but is rather randomly distributed into the lipid matrix, acting as an inert spacer between DPPC molecules.

Further investigations of the interactions of PZQ with phospholipid membranes were performed using either liposomes of dipalmitoylphosphatidylglycerol (DPPG) or dipalmitoylphosphatidylcholine (DPPC). Both phospholipids obtain the same fatty acid, but the polar head groups are different with respect to electrical charge (Harder et al. 1980; Harder et al. 1983): DPPG is an acidic phospholipid with a negatively charged polar head group, while DPPC contains an electrically neutral polar head group. Therefore, liposomes of DPPC or DPPG are ideal to investigate hydrophobic effects of PZQ and effects of Ca^++^ ions. The bilayered structure of DPPG is not disturbed in presence of PZQ (molar ratio DPPG: PZQ 1:1). However, further addition of Ca^++^ ions leads to an instantaneous precipitation with complete resolution of bilayered structures. Thus, PZQ in combination with Ca^++^ ions exert deleterious destruction of the organization of formerly DPPG bilayered membranes. This effect was not observed with corresponding DPPC membranes. Therefore, hydrophobic interactions of PZQ with fatty acids of DPPG and simultaneous interactions of Ca^++^ ions with the negatively charged polar head group of this phospholipid may explain the resolution of bilayered structures of DPPG and induction of fusion processes.

To further study possible structural alterations of synthetic phospholipids in presence of PZQ, ^31^P-nuclear magnetic resonance spectroscopy has been used. This technique allows the study of the effect of drugs, ions, and temperature on the transition between three different phases that a phospholipid can adopt: (1) the bilayer phase, in which biological membranes are normally organized, (2) the isotropic phase (vesicles, inverted vesicles, micellar, cubic, or rhombic structures), and (3) the hexagonal (HII) phase (Allen et al. 1990; Jouhet 2013). The HII phase consists of hexagonically packed lipidic cylinders in which the polar headgroups are oriented toward an internal aqueous channel of nearly 20 Å diameter. This hexagonal organization of lipid molecules cannot constitute a permeability barrier which is otherwise well maintained by bilayered membranes (Cullis et al. 1985a; Cullis et al. 1985b). The formation of HII structures is known to facilitate fusion processes of membranes, which are involved in, e.g., secretion or modulating membrane-associated enzyme activities (Jouhet 2013). In addition, cells adjust their membrane lipid composition in response to perturbation in order to maintain bilayer stability by keeping the bilayer close to a point of instability, where a confined transformation to some non-bilayer structure would tend to occur (Jouhet 2013).

**Fig. 1:**
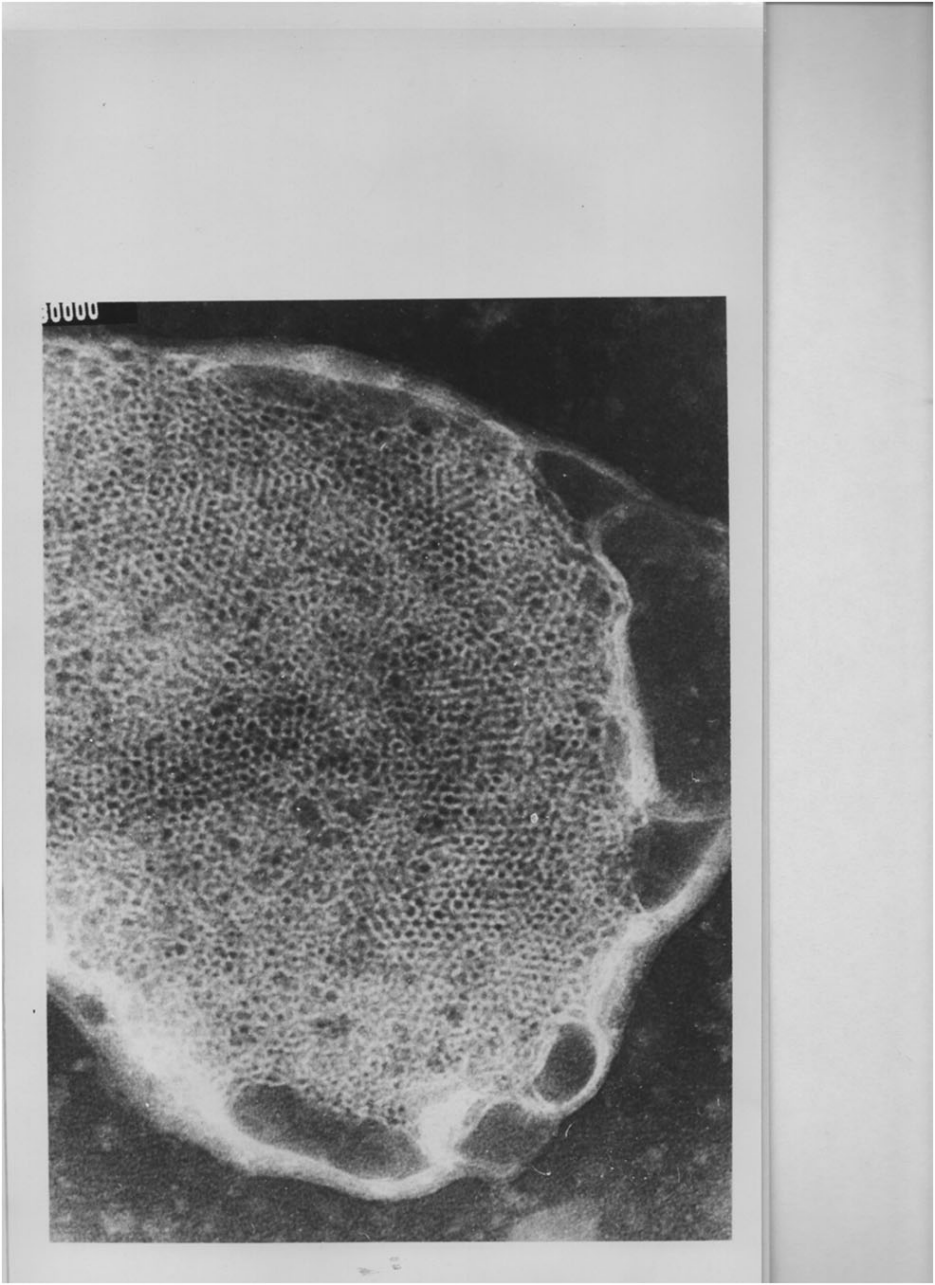
Electron microscopic picture (unpublished) of tubules of the HII phase of a mixture of egg-yolk phosphatidylethanolamine, bovine brain phosphatidylserine, praziquantel, and Ca^++^ (molar ratios PE+PS/PZQ 2:1, PS/Ca^++^ 1:1). Magnification: 250.000-fold, 2.5 cm equals 0.1 μm). This transition is nearly complete showing inverted tubules with the fatty acid chains on the outside of the tubules and polar head groups in the center forming aqueous channels

A phospholipid dispersion consisting of only PE (egg-yolk phosphatidylethanolamine) and PS (bovine brain phosphatidylserine) (molar ratio PE/PS 2:1), which corresponds to the assumed phospholipid composition of tegument lipids in schistosomes, gives rise to a mixture of isotropic and bilayer signals, i.e., there is a coexistence of isotropic and bilayer phases (Harder et al. 1988). In presence of only Ca^++^ ions (molar ratio PE/PS/Ca^++^ 2:1:1), there is only a bilayer phase present. Addition of PZQ alone results in formation of a pure isotropic phase (molar ratio PE + PS/PZQ 2:1). However, if PZQ and Ca^++^ ions are present simultaneously (molar ratios PE + PS/PZQ 2:1; PS/Ca^++^ 1:1), a transition to the HII phase occurs (Harder et al. 1988). This transition is nearly complete showing inverted tubules with the fatty acid chains on the outside of the tubules and polar head groups in the center forming aqueous channels (Fig. 1).

The transitions from bilayer to the HII phase are dependent on lipid to Ca^++^ ratios and on PE: PS ratios and are also temperature dependent. For example, there is only partial transition to the hexagonal HII phase at 25 °C but a nearly exclusive transition to this phase at 35 °C (Harder et al. 1988).

As the HII phase is known to be associated with fusion processes (Jouhet 2013), it is possible that this could also hold for the natural condition. However, until now there is no direct proof that similar transitions actually occur in natural schistosome membranes. Electron microscopy after freeze fracture preparation of schistosome membranes may reveal such changes in the schistosome tegument.

Noteworthy, concentrations of PZQ as low as 3 μM (equivalent to a lipid: PZQ ratio 10:1) have still resulted in the formation of HII phases in presence of Ca^++^ ions (Harder et al. 1988). This is in agreement with the threshold concentration in serum of 3.2 μM at which blebs and vacuolization processes are observed in the tegument of schistosomes (Andrews 1985).

Now a crucial question arises, whether the PZQ-induced perturbations in synthetic phospholipid membranes in presence of Ca^++^ are stereospecific. Indeed, we could not observe any differences between (R)-PZQ and (S)-PZQ in inducing the transition from bilayer or isotropic to HII phases. Therefore, the PZQ-induced transition from bilayer to HII phases in presence of Ca^++^ is not stereoselective and therefore does not explain the mechanism of action of PZQ by itself. The lack of stereoselectivity makes it imperative to postulate the involvement of PZQ receptor protein(s) in mode of PZQ action.

## PZC receptor proteins

A variety of different targets have been suggested to be involved in the PZQ’s mode of action. These are as follows: ATPase of *S. mansoni* (Nechay et al. 1980) or different phosphatases of the cestode *Cotugnia digonophora* (Pampori 1985). These observations may be explained more by indirect PZQ actions as secondary effects caused by severe tegument disruptions.

Angelucci et al. (2007) investigated PZQ impaired adenosine or uracil uptake in living schistosomes. Interestingly, this effect was stereoselective as only (R-)-PZQ was active. There might be a possible relationship between inhibition of nucleoside uptake and the action on Ca^++^ channels by PZQ, since adenosine is able to bind to specific receptors and to function as an indirect antagonist of Ca^++^ release in mammalian cells. However, this hypothesis has not been proven in schistosomes (Angelucci et al. 2007).

Regulatory myosin light chain (MLC) of *S. mansoni* was discussed as another PZQ target (Munirathinam et al. 2009). Using molecular biological tools, these authors identified a clone that strongly binds PZQ and which coded for the MLC protein. As PZQ also affects MLC function in *S. mansoni*, this protein was concluded to play a role in PZQ action.

Pica-Mattocia et al. (2007) tested the hypothesis if Ca^++^ channels of schistosomes are targets of PZQ action. In the presence of Ca^++^ channel blockers nicardipine and nifedipine, schistosomes survived at PZQ concentrations of 3 μM which are normally lethal to adult male worms. In addition, the actin depolymerizing agent cytochalasin D rendered schistosomes completely refractory to high PZQ concentrations up to 36 μM. As the organization of actin cytoskeleton controls Ca^++^ channel activities, these effects support the ideas that schistosomal Ca^++^ channels are involved in the mechanism of action of PZQ (Troiani et al. 2007; Pica-Mattocia et al. 2007). However, this view remains questionable since there is a lack between PZQ-induced intra-worm Ca^++^ influx and parasite death (Pica-Mattoccia et al. 2008).

At present, research on the PZQ action is mainly focused on three targets: (1) cellular Ca^++^ channels like Sm.TRPM_PZQ_ (and homologs) (Park et al. 2019a), whereby PZQ evokes disruption of Ca^++^ homeostasis in the trematodes *S. mansoni*, *S. japonicum*, *S. haematobium*, or *Clonorchis sinensis* and cestodes *Taenia solium*, *Echinococcus multilocularia*, or *Hymenolepis microstoma* (Chan et al. 2013). (2) A specific host serotoninergic G protein-coupled receptor (GPCR) 5-HT_2B_R, which exhibits stereoselective interactions with (R)-PZQ (Park and Marchant 2020). PZQ acts as a low-affinity partial agonist at 5-HT_2B_R. (3) There are several transient receptor potential (TRP) Ca^++^ channels either in mammals or in schistosomes which are activated stereoselectively by PZQ (Chan et al. 2017; Park and Marchant 2020).

One of these host TRP channels hTRPM8 is involved in PZQ-induced relaxation of mesenteric arteries, the site where *S. mansoni* adults reside (Gunaratne et al. 2018). Interestingly, this effect is caused by (S)-PZQ and not by the anthelmintically more active (R)-PZQ (Park and Marchant 2020).

TRP channels are members of a diverse family of cation channels. They are expressed in excitable as well nonexcitable cells and play various important roles in cell signaling and sensory physiology (reviewed by Park and Marchant 2020). They are non-selective cation channels permeable to Ca^++^, Na^+^, and/or K^+^. They are inhibited by Mg^++^ or La^+++^, but not by nifedipine, which blocks voltage activated Ca^++^ channels.

The schistosome-specific transient receptor potential (TRP) channel Sm.TRPM_PZQ_ belongs to the largest of the six subfamilies called transient receptor potential melastatin (TRPM) channels. There are eight distinct TRPM channels in humans (TRPM1-TRPM8), which can be activated by various ligands, temperature, or voltage, and they exhibit a wide range of permeabilities to Ca^++^ (reviewed by Park and Marchant 2020). The Sm.TRPM_PZQ_ channels presumably allow Ca^++^ entry through the tegumental membranes into schistosomes and into their muscle cells followed by contraction. (R)-PZQ stereoselectively activates Sm.TRPM_PZQ_ thus enhancing Ca^++^ permeability (Park et al. 2019b; Park et al. 2019a; Park and Marchant 2020). This TRP channel is present in schistosomes and other PZQ-sensitive parasites (flukes and cestodes). Sm.TRPM_PZQ_ can be regarded as the target protein of PZQ for three reasons: (1) it stereoselectively binds PZQ, (2) the EC_50_ of nearly 600 nM for PZQ activation of Sm.TRPM_PZQ_ is in full agreement with threshold concentrations at which PZQ induces spastic paralysis and schistosome tegument damages, and (3) it is present in a variety of different PZQ-sensitive trematodes and cestodes.

The differences between the human GPCR targets and TRP channels and the schistosome TRP channel are due to the highly significant differences in EC_50_ values. The EC_50_ value for (R)-PZQ as a low-affinity partial agonist of human 5-HT_2B_R is about 8 μM. (S)-PZQ activates TRPM8 with an EC_50_ of about 20 μM. By contrast, (R)-PZQ activates Sm.TRPM_PZQ_ of *S. mansoni* with an EC_50_ of about 150 nM (Park and Marchant 2020). These differences in EC_50_ values of PZQ activation of host 5-HT_2B_R and hTRPM8 channel reflects the selective action of the drug against schistosomes and may explain the good tolerability of PZQ in human and veterinary use (Andrews et al. 1983; Andrews 1985; Cupit and Cunningham 2015) and may give an explanation why PZQ does not influence the integrity of host membranes at therapeutic doses against schistosomes.

## The fate of schistosomes, other trematodes, and cestodes in host animals after PZQ treatment

The fate of schistosomes in mice after PZQ treatment at curative doses is as follows (Andrews 1985): the tegument blebbing is followed within minutes by increasing vacuolation and by formation by larger balloon-like surface exudates, which still contain tegument membranes. A total of 15 min after treatment, worms are found contracted, paralyzed, and translocated from the mesenteric veins to the liver. Previously unexposed parasite antigens are exposed within 30 min in mice and induce complement activation (Skelly 2004). Four hours after PZQ treatment, granulocytes are attached to the schistosomes. Morbid worms become fixed to the walls of blood vessels by fibroblasts. Seventeen hours after treatment, phagocytic cells have invaded parasites resulting in lysis of the parasitic tissues within 7 days. Finally, the dead worms disintegrate rapidly, enclosed in a granulomatous reaction in the liver within 14–18 days after treatment (Mehlhorn et al. 1981).

The fate of intestinal cestodes after PZQ treatment is as follows: after strong contraction and paralysis within 5–10 min, tegument vacuoles are visible in the neck regions of *Hymenolepis nana* after 5 min, and, in addition, teguments became distended (*H. nana*, *H. diminuta*). After 8 min, paralyzed *H. microstoma* worms were no longer attached to the wall of the bile duct; in *Diphyllobothrium latum*, worms strongly contracted were expelled within 30–35 min (Chai 2013). PZQ-evoked paralysis and tegument disrupture is also seen in intestinal trematodes, and worms are expelled from the intestine (Chai 2013). Gut immune reactions and/or digestive enzymes may also play a role in further maceration of worms.

## Model for the mechanism of action of praziquantel

**Fig. 2:**
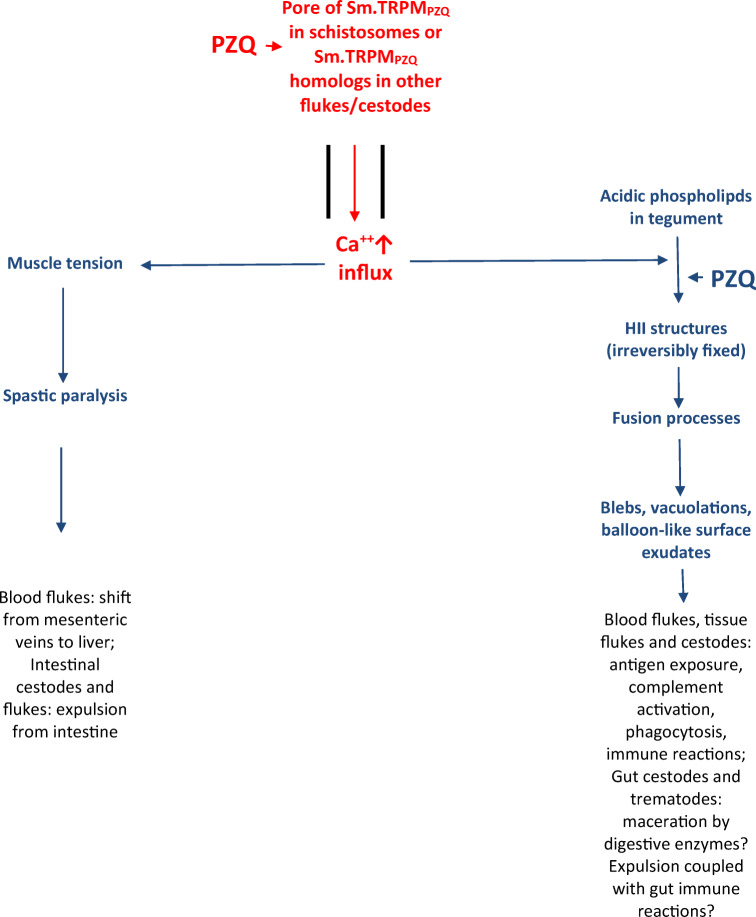
Schematic diagram of the action of PZQ in *S. mansoni*, other trematodes, and cestodes. PZQ activates the transient receptor potential (TRP) channel Sm.TRPM_PZQ_ in *S. mansoni* with an EC_50_ about 150 nM (Park and Marchant 2020). This results in a highly increased Ca^++^ influx, followed by perturbation of Ca^++^ homeostasis within worms (red). For PZQ-sensitive flukes and cestodes, the presence of Sm.TRPM_PZQ_ homologs is postulated. Activation of this channel by PZQ accompanied by high Ca^++^ influx leads to increase in muscle tension ending with spastic paralysis and simultaneously formation of HII structures in the tegument membranes (blue). The latter is mediated by the simultaneous action of Ca^++^ and PZQ and is an irreversible process (Harder 2013). These then initiate fusion processes, formations of blebs, vacuolations, and large balloon-like surface exudates (blue). These processes are visible in all PZQ-sensitive flukes and cestodes Andrews 1985, Chai 2013). In tissue flukes, blood flukes, and cestodes, previously unexposed parasite antigens now become exposed and induce a cascade of immune reactions involving complement activation and attachment of immune cells (e.g., granulocytes) (black). In the case of schistosomes, morbid worms are fixed to walls of blood vessels by fibroblasts, and phagocytic cells invade parasites ending in lysis of their tissues, rapid disintegration of dead worms, and enclosure in granulomas in the liver (Mehlhorn et al. 1981). Also, intestinal cestodes and trematodes become quickly paralyzed and their tegument disrupted by PZQ before they are expelled from the intestine (Chai 2013). Here gut immune reactions and/or digestive enzymes may also play a role

According to the model of PZQ action in schistosomes, PZQ activation of Sm.TRPM_PZQ_, Ca^++^ influx, muscle paralysis, and tegument disruption are tightly coupled with each other. All three events take place in an overall PZQ concentration range of 0.1–1 μM within the first minute after drug exposure (Table 1).

(R)-PZQ stereoselectively activates Sm.TRPM_PZQ_, a transient receptor potential channel in a nanomolar range (Park et al. 2019b; Park and Marchant 2020). This is followed by Ca^++^ influx in to schistosomes (Park and Marchant 2020). As a result, Ca^++^ homeostasis becomes disturbed within the worms. Thereafter, two Ca^++^-dependent processes are initiated: spastic paralysis and tegument disruption (Fig. 2).

A model for the PZQ-induced tegument disruption is based on the presence of domains in the double bilayered surface membranes (Jouhet 2013). These domains in the tegument membranes elicit different responses to PZQ reflected by different fluidity behaviors (Lima et al. 1994a, 1994b) and specific membrane compositions (Redman et al. 1996).

Ca^++^ and PZQ, which is already present in the phospholipid phase of the membrane (Fig. 2), come into close contact with each other. While Ca^++^ interacts only with negatively charged polar head groups of phospholipids like phosphatidylserine (PS) or phosphatidylglycerol (PG), the electrically neutral lipophilic PZQ interacts with the lipophilic phospholipid phase of all membrane phospholipids (Schepers et al. 1988; Harder 2013).

The interactions of PZQ, Ca^++^, and negatively charged phospholipids, which are not stereoselective, take place in specific membrane domains and induce stable, irreversible HII structures, followed by severe perturbations of bilayer structures. Thus, the balance between bilayer, isotropic, and HII phases which is characteristic of natural membranes (Jouhet 2013) becomes disturbed by PZQ and Ca^++^. Moreover, HII structures are the sites of fusion processes within membranes (Jouhet 2013). Because of irreversible fixation of HII structures by PZQ and Ca^++^, production of blebs and vacuolations are initiated followed by complete perturbations of schistosome tegument membranes, undirected Ca^++^, K^+^, and Na^+^ influxes, and disturbed integrity of normal enzyme functions.

Altogether, the combined involvement of Sm.TRPM_PZQ_ and Ca^++^ influx in PZQ action, followed by muscular paralysis and appearance of tegument disrupture, induces a lethal cascade in schistosomes. This deadly cascade is perhaps also operating in trematodes other than schistosomes and in cestodes since homologs of Sm.TRPM_PZQ_ could be detected in *S. japonicum*, *S. haematobium*, or *Clonorchis sinensis* and cestodes *Taenia solium*, *Echinococcus multilocularia*, or *Hymenolepis microstoma* (Park et al. 2019a).

## Outlook

The urgency to look for new antischistosomal and anticestodal compounds is supported by the recent alarming identification of PZQ resistance of the zoonotic cestode *Dipylidium caninum*, which infects dogs, cats, and humans (Chelladurai et al. 2018. Thus, clinical resistance was detected in dogs against PZQ and the structurally related epsiprantel. There seems to be a beginning of resistance problems in veterinary medicine that may become more serious in the future. This should be a stimulus for clarifying the mechanism of PZQ resistance.

As host receptors 5-HT_2B_R, TRPM8, or Sm.TRPM_PZQ_ of *S. mansoni* are targets of PZQ action, they will be possibly valuable tools for detection of new lead structures for new antischistosomal, other antitrematodal, and anticestodal compounds (Nogi et al. 2009; Zhang et al. 2011; Chan et al. 2014; Chan et al. 2015; Chan et al. 2016; Chan et al. 2018, Park and Marchant 2020). Some of the lead structures and compounds seem to look promising which might replace PZQ in the future.

Further research should also focus on the characterization of the binding site of PZQ at Sm.TRPM_PZQ_ of *S. mansoni* or other PZQ-sensitive flukes and cestodes. More than 400 PZQ derivatives have been tested; the structures of the most important are given in Andrews et al. 1983. Beyond these derivatives, there are some which elicit high anthelmintic activities, e.g., para-aminobenzyl derivative, and there are also some without any activities. This could be a basis for intensified investigation of structure-activity relationships and for optimizing the structure of PZQ by molecular modeling at Sm.TRPM_PZQ_ of *S. mansoni* or homolog channels.

Interestingly, HII structures may be important for some enzyme activities. The activities of the Ca^++^ pump in the sarcoplasmic reticulum, the CTP:phosphocholine cytidyltransferase, or the violaxanthin de-epoxidase in thylakoids are dependent on HII structures; membrane anchoring of some proteins like G proteins and phosphokinases C is enhanced by HII phase (reviewed by Jouhet 2013). Is it possible that also Sm.TRPM_PZQ_ of *S. mansoni* or homolog channels are associated with HII structures?

With respect to PZQ resistance in *Dipylidium caninum*, an investigation into the mechanism of PZQ resistance could give a hint to look for any structural alterations of Sm.TRPM_PZQ_ homolog in this parasite.
